# Admission homocysteine is an independent predictor of spontaneous reperfusion and early infarct-related artery patency before primary percutaneous coronary intervention in ST-segment elevation myocardial infarction

**DOI:** 10.1186/s12872-018-0868-3

**Published:** 2018-06-25

**Authors:** Jing Li, Ying Zhou, Yaowen Zhang, Jingang Zheng

**Affiliations:** 1 Department of Cardiology, China-Japan Friendship Hospital, No. 2, Yinghua Road, Beijing, 100029 China; 2 Medieco Group Co. Ltd, B901 Building No.20 Hepingxiyuan, Beijing, 100029 China

**Keywords:** ST-segment elevation myocardial infarction, Spontaneous reperfusion, Pre-interventional IRA patency, Predictor, Homocysteine

## Abstract

**Background:**

Spontaneous reperfusion (SR) and early infarct related artery (IRA) patency before primary percutaneous coronary intervention (PPCI) might bring extra benefit for patients with ST-segment elevation myocardial infarction (STEMI). This study premilinarily screened the independent predictors of SR, and assessed the relationship between SR and plasma homocysteine (HCY).

**Methods:**

The medical records of 998 patients who were diagnosed as STEMI and underwent emergency coronary angiography were retrospectively studied, SR was defined as achievement of TIMI grade 3 flow in the IRA before PCI. The baseline characteristics, clinical manifestations and hematological variables were compared between SR and NSR group. Optimal cutoff point of HCY was calculated with receiving operating characteristics (ROC) analysis, multivariate logistic regression models were used to identify predictors of SR.

**Results:**

229 (22.95%) patients showed angiographic SR. For HCY, the area under the curve was 0.70 (95% CI: 0.63–0.77, *P* = 0.034), the optimized cut off point was 17.55 μmol/L. Preinfarct angina (95% CI: 1.61–5.65, *P* = 0.0005), plasma C-reactive protein (CRP) level (95% CI: 0.87–0.99, *P* = 0.016) and HCY < 17.55 μmol/L (95% CI: 2.43–8.72, *P* < 0.0001) were found to be independent predictors for SR.

**Conclusion:**

In patients with STEMI, HCY < 17.55 μmol/L, preinfarct angina and plasma CRP level were independent predictors of SR.

## Background

Early infarct related artery (IRA) patency and prompt myocardial reperfusion are crucial for improving clinical outcomes in patients with ST-segment elevation myocardial infarction (STEMI) [1], primary percutaneous coronary intervention (PPCI) is the current therapy of choice to achieve IRA patency. Furthermore, outcomes of patients with STEMI undergoing PPCI are closely related to the initial IRA blood flow prior to PPCI [2–4]. In an analysis from the Primary Angioplasty in Myocardial Infarction (PAMI) trials [5], initial thrombolysis in myocardial infarction (TIMI) grade 3 flow derived from spontaneous reperfusion (SR) was associated with improved left ventricular function, decreased adverse cardiovascular events and reduced short and long-term mortality independent of successful PPCI, implying that SR might bring extra benefit of myocardial salvage for STEMI patients before PPCI. Thus the concept of IRA patency was extended to the pre-PPCI phase, to predict and facilitate SR might provide alternative methods for acute risk assessment and tailoring of adjunctive therapies during coronay intervention, predictors of SR might thus provide important prognostic information. However, little has been known about the predictors or clinical biomarkers SR.

Since thrombus formation is the main mechanism of the obstruction of IRA in STEMI [6], and timely spontaneous thrombolysis lead to spontaneous reperfusion [7], factors influencing spontaneous thrombolysis might be able to predict the process of SR.

Homocysteine (HCY) is a sulfur-containing amino acid that functions as a key intermediate in methionine metabolism [8], which is believed to promote atherothrombosis through several mechanism [9], and hyperhomocystenemia (HHCY) (plasma HCY > 10 mmol/L) is currently recognized as a new independent risk factor for atherosclerotic vascualr disease. Furthermore, HHCY is related to impaired formation of the fibrin networkby inducing slower coagulation process and rendering more tightly packed fibrin clots, hence influencing the process of spontaneous thrombolysis [10]. Therefore, we speculated that HCY might be a predictor of early SR in STEMI patients.

In the present study, we aimed to premilinarily screen the clinical predictors of SR and assess the possible relationship between SR and plasma HCY.

## Methods

### Patient population

This retrospective, single center observational study was based on review of medical records, electrocardiographic analysis and cardiac catheterization films of 998 consecutive patients presenting from January 1,2012 through January 1,2015 to China-JapanFriendshipHospital (Beijing, China) with suspected acute STEMI, who underwent emergency coronary angiography on admission within 12 h of symptom onset and activation of the emergency PCI protocol. Exclusion criteria were: coronary spasm proved to be responsible for STEMI with angiographic SR; left bundle branch block; thrombolytic therapy before arrival to our hospital; and final diagnosis other than STEMI, such as non-ST-segment elevation myocardial infarction (NSTEMI), unstable angina, takotsubo cardiomyopathy and myocarditis.

Diagnosis of STEMI was based on the presence of symptoms of ischemia, increased serum biomarkers (cardiac-specific troponin) and ST-segment elevation≥1 mm in ≥2 contiguous leads [11]. Angiographic SR was defined as achievement of TIMI grade 3 flow in the IRA before PCI (first contrast injection) [6].

### Laboratory analysis

In all patients, venous peripheral blood samples were taken on admission for measurement of hematological variables. Samples were obtained in the emergency room or coronary care unit (CCU) before medication, and processed within 30 min from collection, the first dataset was used for analysis if more than one assessment was performed. Blood counts were measured by the BECKMAN COULTER LH780 Hematology analyzer system (USA). Plasma creatinine, uric acid, HCY and C-reactive protein were measured by BECKMAN COULTER Chemistry Analyzer AU5800 (USA). Prothrombin Time and Activated partial thromboplastin time were measured by a STAGO Compact (France). Troponin-I was measured by a BECKMAN Image 800 Analyzer (USA).

In all patients, the left ventricular ejection fraction (LVEF) was measured within 24 h since admitted in emergency room by tracing contours of the LV using the manual biplane Simpson’s rule [12](Vivid E9, GE Ultrasound A5, Horten, Norway).

### Medication during hospital stay

All patients received a loading dose of 300 mg aspirin and 300-600 mg clopidogrel, and a single subcutaneous bolus of low molecular weight heparin (LMWH) in transit or at the emergency room. 3000 U intravenous heparin was routinely administered in the catheterization room before angiography. An additional dose of (6000 U) and glycoprotein IIb/IIIa antagonist would be adopted if PPCI was necessary. All patients were managed with standard administration of 100 mg aspirin, 75 mg clopidogrel, ß-blockers, ACEI/ARB and statins according to the guidelines for STEMI.

### Coronary angiography

All patients received emergency angiography within 12 h of symptom onset using standard techniques. IRA patency referred to those with TIMI grade 3 flow. Spontaneous reperfusion referred to angiographic SR described in “patient population” section. Time to angiography was defined as the time from onset of ischemic symptoms to the first contrast injection. According to the European Society of Cardiology (ESC) guidelines (2012), primary PCI is defined as PCI in patients with STEMI within 12 h of the onset of chest pain [13].

### Statistical analysis

Statistical analyses were performed using SPSS 22.0 software (SPSS Inc., Chicago, USA). Continuous variables were presented as mean ± standard deviation (SD) for normally distributed data, or as median (interquartile range, IQR) for non-normally distributed data, and compared with the Student’s T test. Non-parametrics tests were used for non-normally distributed data. Categorical variables were expressed as percentages (%) and compared using the chi-square test.

Optimal cutoff point of HCY was calculated with receiving operating characteristics (ROC) analysis. All *P* values referred to 2-tailed tests of significance, and *P* < 0.05 was considered significant. HCY was transferred into binary variable according to the cutoff point.

All variables showing significant differences in the univariate comparison were included in the multiple logistic regression analysisto identify predictors of SR. The candidate variables entering multiple logistic regression analysis were: preinfarct angina, previous long-term aspirin medication, uric acid level, CRP level, NLR, PLR and the binary variable of HCY above or below cutoff point. The results were expressed by the odds ratio (OR) and corresponding 95% confidence interval (CI).

## Results

### Baseline characteristics

Among the 998 STEMI patients enrolled, 229 (22.95%) showed angiographic SR, 156 (68%) of the 229 patients showed ST-segment resolution ≥70% (electrocardiographic SR) before angiography. Distributions of baseline, clinical manifestations and revascularization procedures between patients with SR (SR group) and without SR (NSR group) are shown in Table 1. Multivariate logistic regression models adjusted by age, gender, cardiovascular risk factors, antiplatelet therapy, time to angiography and hematological variables were used to identify predictors of SR.There were no significantly differences in terms of age, gender, family history of myocardial infarction (MI), major cardiovascular risk factors such as current smoker, hypertension, diabetes mellitus, dyslipidemia and chronic renal failure, history of MI, or long-term (>one year) medication of statin between groups. Frequencies of previous long-term aspirin medication and preinfarct angina were higher in the SR group than in the NSR group (*P* < 0.05, respectively). During hospitalization, there were no differences in medication of aspirin, clopidogrel, heparin or GP IIb/IIIa antagonists between the groups. Hematological variables on admission are shown in Table 2. Compared with the NSR group, plasma HCY, CRP, uric acid, NLR and Platelet/lymphocyte ratio (PLR) levels were significantly lower in SR group (*P* < 0.05, for all).

**Table 1:** Baseline characteristics of study patients

	SR	NSR	*P* Value
	(*n* = 229)	(*n* = 769)	
Clinical characteristics			
Age(years)	62.13 ± 11.27	61.79 ± 12.86	0.877
Male sex, n (%)	186 (81.22)	636 (82.70)	0.605
Current smokers, n (%)	132 (57.81)	454 (59.11)	0.706
^a^Family history, n (%)	32 (13.97)	89 (11.57)	0.329
Hypertension, n (%)	113 (49.34)	424 (55.14)	0.122
Diabetes, n (%)	46 (20.31)	185 (24.14)	0.211
Stroke, n (%)	25 (10.92)	93 (12.09)	0.628
Dyslipidemia, n (%)	128 (55.89)	414 (53.84)	0.583
Chronic renal failure, n (%)	10 (4.37)	29 (3.77)	0.683
^b^Previous revascularization, n (%)	11 (4.80)	67 (8.71)	0.053
Previous long-term statin, n (%)	36 (15.72)	91 (11.83)	0.121
Previous long-term aspirin, n (%)	57 (24.89)	143 (18.59)	0.037
Preinfarct angina within 1 month, n (%)	143 (62.45)	293 (38.10)	0.001
Time to angiography, hours	5.1[2.7, 7.8]	4.3[2.6, 7.2]	0.212
Clinical manifestations			
SBP, mmHg	124.25 ± 23.07	117.41 ± 21.56	0.018
DBP, mmHg	76.51 ± 12.59	75.44 ± 12.12	0.067
Hear rate, beats/min	67.41 ± 11.1	73.12 ± 14.69	0.021
LVEF, %	58 ± 11	49 ± 10	0.001
Killip2–4, n (%)	50 (21.83)	161(20.93)	0.770
Killip3–4, n (%)	21 (7.86)	87 (10.97)	0.359
IABP use, n(%)	8 (3.49)	80 (10.40)	0.001
Infarct location, n (%)			0.203
Anterior	141 (61.57)	429 (55.79)	
Inferior	78 (34.06)	312 (40.57)	
Lateral	10 (4.37)	28 (3.64)	
Medication of antithrombotic drugs during hospitalization			
Aspirin	229 (100%)	769 (100%)	N/A
Clopidogrel	229 (100%)	769 (100%)	N/A
GP-IIb/IIIa antagonists	44 (19%)	130 (17%)	0.42
Heparin	229 (100%)	769 (100%)	N/A
Revascularization procedures, n (%)			
Primary PCI	145 (63.31)	710 (92.32)	< 0.001
No revascularization	69 (30.13)	59 (7.67)	< 0.001

Data are presented as mean ± SD, IQR or number (percentage)

^a^Family history referred to the history of acute myocardial infarction of the patients’ parents, brothers or sisters

^b^PCI or coronary artery bypass grafting

**Table 2:** Baseline hematological variables of study patients

	SR	NSR	*P* Value
	(*n* = 229)	(*n* = 769)	
Mean platelet volume, fL	10.69 ± 1.37	10.74 ± 0.83	0.787
Red cell distribution width, fL	41.4 ± 2.26	41.76 ± 3.04	0.317
Hemocysteine, μmol/L	11.9[9.1, 16.7]	17.8[12.2, 21.8]	<.0001
Peak Troponin-I, ng/mL	8.89[0.76, 15.75]	19.91[8.63, 30.0]	<.0001
Creatinine, umol/mL	81.45[73, 92]	77 [67, 91]	0.101
C-reactive protein,mg/dL	3.48[2.2, 6.35]	5.48[3, 10.1]	0.010
Prothrombin Time, second	13.2[12.6, 13.6]	13.3[12.7, 14.1]	0.159
Activatedpartialthromboplastin time, second	37.85[35.25, 44.65]	40.15[34.8, 48]	0.457
Neutrophil count, × 10^9^/L	210.34[184.50, 235.50]	216.38[182.50, 250.0]	0.210
Leukocyte count, ×10^9^/L	6.31[4.22, 7.57]	7.13[5.21, 8.47]	0.030
Platelet count, ×10^9^/L	2.10[1.5, 2.4]	1.90[1.28, 2.38]	0.080
Neutrophil/lymphocyte ratio	2.86[2.06, 4.18]	3.58[2.58, 5.41]	0.005
Platelet/lymphocyte ratio	108.33[82.7, 138.1]	121.2[90.59, 169.11]	0.053
Creatinine> 108 umol/L, n (%)	14(21.88)	46(22.66)	0.896
Uric acid> 420 mmol/L(male) or > 380(female), n (%)	20(31.25)	33(16.26)	0.009

### Receiver operating characteristic (ROC) curve analysis of HCY

**Fig. 1:**
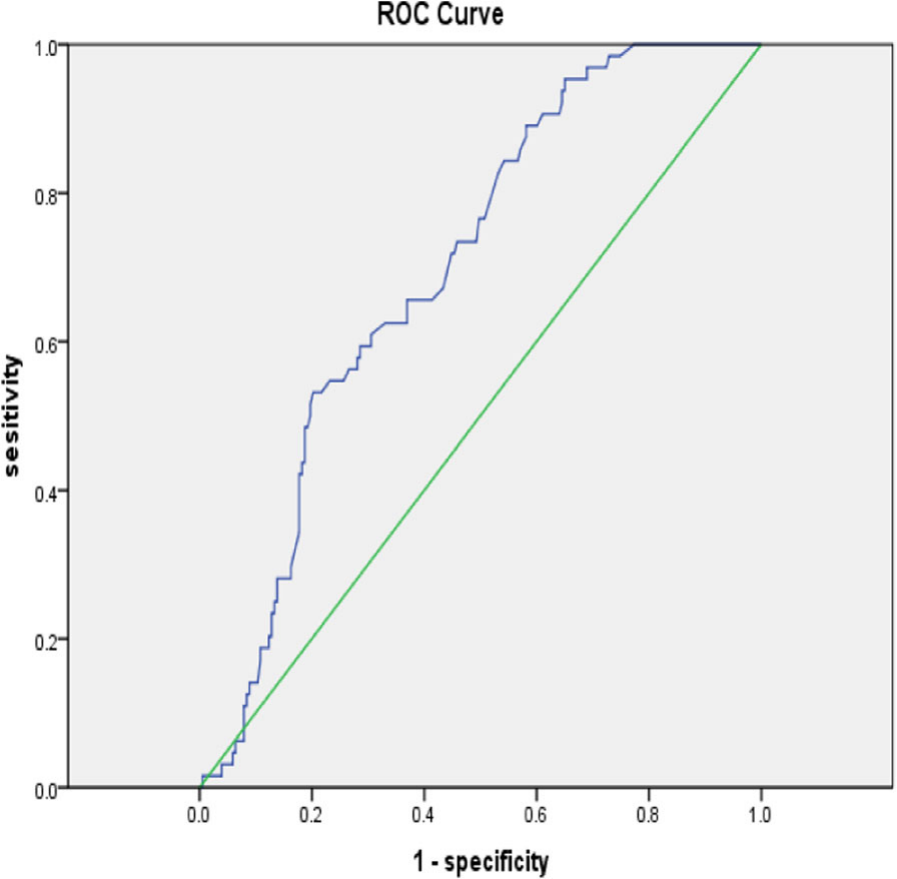
ROC curve for HCY levels in patients with SR in the baseline angiography. The mean area under the ROC curve was 0.70.

The value of HCY as predictors of SR was evaluated by means of ROC analysis (Fig. 1).

For HCY, the area under the curve was 0.70 (95% CI: 0.63–0.77, *P* = 0.034). An optimized cutoff point of 17.55 μmol/L showed a sensitivity of 0.53 and specificity of 0.20 for prediction of SR. HCY < 17.55 μmol/L was more common in patients with SR.

HCY was transferred into binary variable according to the cutoff point of 17.55 μmol/L, then HCY ≥17.55 μmol/L and < 17.55 μmol/L as a binary variable, together with preinfarct angina, CRP and NLR were tested by multiple logistic regression to decide the independent predictors of SR.

### Independent predictors of SR in STEMI

Preinfarct angina (95% CI: 1.61–5.65, *P* = 0.0005), plasma C-reactive protein (CRP) level (95% CI: 0.87–0.99, *P* = 0.016) and HCY < 17.55 μmol/L (95% CI: 2.43–8.72, *P* < 0.0001) were found to be independent predictors for SR on multiple logistic regression analysis (Table 3). For these 3 predictors, ROC curve analysis showed the area under the ROC curve was 0.74, 95% CI was 0.68–0.79.

**Table 3:** Multivariable analysis for the independent predictors of SR

	OR	95% Confidence Interval	*P* Value
Preinfarct angina	3.02	1.61–5.65	0.0005
C-reactive protein	0.92	0.87–0.99	0.016
Hemocysteine< 17.55 μmol/L	4.61	2.43–8.72	< 0.0001

*OR* Odds ratio

### In-hospital and 1-year outcomes

The in-hospital and 1-year clinical outcomes are summarized in Table 4.

Patients with SR showed a significantly better in-hospital course with lower in-hospital mortality. Patients with SR had better preserved heart function, with lower rates of congestive heart failure, pulmonary edema and cardiac shock. Patients with SR also showed a lower rate of malignant cardiac arrhythmia, with lower rates of sustained ventricular tachycardia, primary ventricular fibrillation and asystole. The in-hospital rates of reinfarction were similar between groups.

Patients with SR showed more favorable 1-year outcomes with lower rates of heart failure and mortality. Though patients without SR had more frequently underwent PPCI on IRA, they had statistically similar 1-year rates of reinfarction and ischemia-drived target vessel revascularization compared with patients with SR.

**Table 4:** In-hospital and 1-year clinical outcomes

Variable	SR	NSR	*P* Value
	(*n* = 229)	(*n* = 769)	
In-hospital clinical outcome			
Heart failure, n (%)	13 (5.6)	92 (11.9)	0.006
Pulmonary edema, n (%)	9 (3.9)	69 (9.0)	0.01
Cardiac shock, n (%)	3 (1.3)	39 (5.1)	0.01
Reinfarction, n (%)	6 (2.6)	31 (4.0)	0.32
Primary ventricular fibrillation, n (%)	6 (2.6)	66 (8.6)	0.002
Sustained venticular tachycardia, n (%)	2 (0.8)	34 (4.3)	0.01
High-degree atrioventricular block, n (%)	10 (4.4)	41 (5.3)	0.56
Asystole, n (%)	1 (0.4)	35 (4.6)	0.003
Major bleeding, n (%)	2 (0.9)	12 (3.1)	0.44
All-cause mortality, n (%)	1 (0.4)	21 (2.4)	0.03
1-year clinical outcome			
Reinfarction, n (%)	8 (3.5)	29 (3.8)	0.84
Ischemia-drived target vessel Revascularization, n (%)	25 (10.9)	77 (10.0)	0.69
Heart failure, n (%)	13 (5.6)	107 (13.9)	< 0.001
All-cause mortality, n (%)	2 (0.8)	31 (4.0)	0.02

## Discussion

Our study showed a 22.95% incidence of angiographic SR in patients with STEMI, similar to the incidence of 14%~ 22% in previous studies [5, 14].

In our study, HCY < 17.55 μmol/L, together with preinfarct angina and CRP were proved to be independent predictors of SR in STEMI patients; among the 3 predictors, HCY < 17.55 μmol/L showed the most obvious statistical significance.

To the best of our knowledge, this is the first study that investigated the relationship between HCY and pre-PPCI IRA patency in patients with STEMI. Urgent restoration of blood flow in IRA and early myocardial reperfusion is related to improved survival in patients with STEMI [15].This concept was extended to the pre-PCI phase in an analysis from the Primary Angioplasty in Myocardial Infarction (PAMI) trials [5]. Initial TIMI grade 3 flow remained independently associated with better survival even after adjusting for post-PCI flow. Brener SJ et al. [16] analyzed the combined databases of the Controlled Abciximab and Device Investigation to Lower Late Angioplasty Complications (CADILLAC) and the Harmonizing Outcomes With Revascularization and Stents in Acute Myocardial Infarction (HORIZONS-AMI) trials, suggested that spontaneous reperfusion before PCI might reduce 1-year mortality after primary PCI in STEMI by 39%, even after adjusting for post-PCI TIMI flow. Thus SR could bring extra benefit for the patients with STEMI independent of PPCI. Recognition of non invasive predictors of pre-PPCI SR will provide new treatment information for the very early stage of STEMI, and bring prognostic information.

Because thrombotic occlusion of coronary artery upon a background of atherosclerotic plaque rupture is the ultimate step in the pathogenesis of MI [17], and the fate of an evolving thrombus is largely determined by the balance between coagulating system and fibrinolysis system [18], factors that influence thrombosis and endogenous thrombolysis might play an important role in early SR. The spontaneous lysis of platelet-rich thrombus is an important defense mechanism against lasting coronary occlusion, MI could be regarded as a result of the failure of timely spontaneous thrombolysis/fibrinolysis; on the contrary, rapid enhanced endogenous fibrinolysis could spontaneously dissolve thrombus and lead to SR and IRA patency. As a support of the above hypothesis, Christopoulos C et al. [19] reported that patients with STEMI who demonstrated pre-PPCI SR have enhanced endogenous thrombolysis, decreased platelet reactivity and shorter occlusion time in IRA, implying that factors influencing the balance of thrombosis and endogenous thrombolysis might influence the process of SR, and these factors might become potential markers for SR in patients with STEMI. Plasma HCY level is currently a clinical marker of several atherothrombotic vascular disease, and elevated plasma HCY might have some relation with impaired endogenous thrombolysis, therefore, we speculated that HCY could also be a clinical marker of SR.

HCY is a sulfur-containing amino acid that functions as a key intermediate in methionine metabolism. It is produced as a byproduct of methyl-transfer reactions, which are important for the synthesis of DNA, methylated proteins, neurotransmitters and phospholipids [9]. High plasma level of homocysteine, termed as hyperhomocystenemia (HHCY), has been recognized as a biomarker of atherothrombotic vascular disease, overwhelming clinical and epidemiological studies have identified HHCY(> 15 mmol/L) as a new independent risk factor for athroscleroticvascualr disease [20–24]. Furthermore, HHCY is associated with a greater number of diseased arteries and higher severity of coronary diaseases [25], and might be an important predictor for long-term mortality in patients with acute myocardial infarction (MI) [26].

Angiographic findings demonstrated that HHCY was strongly correlated with slow coronary flow (SCF) phenomenon after PCI protacol [27, 28], implying the possible correlation between HHCY and less IRA patency. Current evidence suggests that the microcirculation dysfunction and damage of endothelial cells caused by HCY-induced oxidative stress played an important role in SCF [29–31]. HCY can reduce the basal production of nitric-oxide (NO) in consequence of the emergence of some biochemically active products such as hydrogen peroxide (H_2_O_2_), superoxide (O_2_^−^) and hydroxyl radical (HO) [32, 33], and enhance NO degradation by inhibiting the synthesis and activity of NO synthase (NOS), thus lead to decreased bioavailability of NO and impair the endothelium-dependant vasodilation [34]. Therefore, the HHCY-induced endothelial dysfunction might explain the worse initial IRA TIMI flow in patients with STEMI.

It has been reported that total HCY plasma levels are associated with clot permeation and susceptibility to fibrinolysis in coronary artery disease [35]. HHCY has been related to impaired formation of the fibrin network [36, 37]. HCY impairs the fibrinolysis networks by inducing slower coagulation process and rendering more tightly packed fibrin clots [10].Under the influence of HCY, fibrin networks resulted in a more compact structure with shorter, thicker and more branched fibers, these structural properties of fibrin are related to slower spontaneous lysis rate of thrombus, and proved to be less permeable and more resistant to fibrinolysis [38, 39]. Moreover, studies have revealed that mild HHCY (>10umol/L) showed markedly relationship with decreased tissue-type plasminogen activator (t-PA) activity (which is the major activator of fibrinolysis) and impaired spontaneous thrombolysis in STEMI patients [40]. Therefore, HHCY might play a negative role in the process of SR by facilitating thrombus formation towards total occlusion anddecreasing the spontaneous thrombolysis/fibrinolysis in IRA.

### Limitations

This is a retrospective, non-randomized design study, we didn’t observe the relationship between HCY and mortality, which may help to further evaluate the prognostic value of HCY. However, the aim of the present study was to preliminarily screening the possible non-invasive, easily available marker of SR, it’s a new idea to assess the relationship of HCY and SR, and this retrospective study had obtained preliminary results which may be a guide for further investigation to evaluate the predictors of pre-PPCI IRA patency.

## Conclusion

The present study showed that in patients with STEMI, preinfarct angina, CRP level, and HCY < 17.55 μmol/L were independent predictors of SR. Elevated plasma level (≥17.55 μmol/L) of HCY was an independent negative predictor of SR. HCY might thus be a useful biomarker to predict early IRA patency in patients with STEMI.

## Abbreviations

CRP: C-reactive protein
HCY: Homocysteine
HHCY: Hyperhomocystenemia
IRA: Infarct-related artery
NLR: Neutrophil/lymphocyte ratio
NO: Nitric-oxide
PCI: Percutaneous coronary intervention
PPCI: Primary percutaneous coronary intervention
SCF: Slow coronary flow
SR: Spontaneous reperfusion
STEMI: ST-segment elevation myocardial infarction
TIMI: Thrombolysis in myocardial infarction

## References

[1] Zeymer U, Huber K, Fu Y (2012). Impact of TIMI 3 patency before primary percutaneous coronary intervention for ST-elevation myocardial infarction on clinical outcome: results from the ASSENT-4 PCI study. Eur Heart J Acute Cardiovasc Care.

[2] Lekston A, Hudzik B, Szkodzinski J (2013). Spontaneous reperfusion before intervention improves immediate but not long-term prognosis in diabetic patients with ST-segment elevation myocardial infarction and multivessel coronary artery disease. Cardiol J.

[3] Christian TF, Milavetz JJ, Miller TD, Clements IP, Holmes DR, Gibbons RJ (1998). Prevalence of spontaneous reperfusion and associated myocardial salvage in patients with acute myocardial infarction. Am Heart J.

[4] Bainey KR, Fu Y, Granger CB (2009). Investigators AA. Benefit of angiographic spontaneous reperfusion in STEMI: does it extend to diabetic patients?. Heart.

[5] Stone GW, Cox D, Garcia E (2001). Normal flow (TIMI-3) before mechanical reperfusion therapy is an independent determinant of survival in acute myocardial infarction: analysis from the primary angioplasty in myocardial infarction trials. Circulation.

[6] Dewood MA, Spores J, Notske R (1980). Prevalance of total coronary occlusion during the early hours of transmural myocardial infarction. N Engl J Med.

[7] Swan HJ (1989). Acute myocardial infarction: a failure of timely, spontaneous thrombolysis. J Am Coll Cardiol.

[8] Selhub J (1999). Homocysteine metabolism. Annu Rev Nutr.

[9] Welch GN, Loscalzo J (1998). Homocysteine and atherothrombosis. N Engl J Med.

[10] Genoud V, Lauricella AM, Kordich LC (2014). Impact of homocysteine-thiolactone on plasma fibrin networks. J Thromb Thrombolysis.

[11] Thygesen K, Alpert JS, Jaffe AS, Simoons ML, Chaitman BR, White HD, Writing group on the joint ESC/ACCF/AHA/WHF task force for the universal definition of myocardial infarction (2012). Third universal definition of myocardial infarction. Eur Heart J.

[12] Lang RM, Bierig M, Devereux RB (2006). Recommendations for chamber quantification. Eur J Echocardiogr.

[13] Steg PG, James SK, Atar D, Task Force on the management of ST segment elevation acute myocardial infarction of the European Society of Cardiology (2012). ESC guidelines for the management of acute myocardial infarction in patients presenting with ST-segment elevation. Eur Heart J.

[14] Fefer P, Hod H, Hammerman H (2009). Relation of clinically defined spontaneous reperfusion to outcome in ST-elevation myocardial infarction. Am J Cardiol.

[15] Investigators TGUSTOA (1993). The effects of tissue plasminogen activator, streptokinase, or both on coronary-artery patency, ventricular function, and survival after acute myocardial infarction. N Engl J Med.

[16] Brener SJ, Mehran R, Brodie BR (2011). Predictors and implications of coronary infarct artery patency at initial angiography in patients with acute myocardial infarction (from the CADILLAC and HORIZONS-AMI trials). Am J Cardiol.

[17] Naghavi M, Libby P, Falk E (2003). From vulnerable plaque to vulnerable patient:a call for new definitions and risk assessment strategies: part II. Circulation.

[18] Bodary PF, Wickenheiser KJ, Eitzman DT (2002). Recent advances in understanding endogenous fibrinolysis: implications for molecular-based treatment of vascular disorders. Expert Rev Mol Med.

[19] Christopoulos C, Farag M, Sullivan K, Wellsted D, Gorog DA (2017). Impaired thrombolytic status predicts adverse cardiac events in patients undergoing primary percutaneous coronary intervention. ThrombHaemost.

[20] Ma Y, Li L, Geng XB (2016). Correlation between hyperhomocysteinemia and outcomes of patients with acute myocardial infarction. Am J Ther.

[21] Wu Y, Huang Y, Hu Y (2013). Hyperhomocysteinemia is an independent risk factor in young patients with coronay artery disease in southern China. Herz.

[22] Rasouli ML, Nasir K, Blumenthal RS, Park R, Aziz DC, Budoff MJ (2005). Plasma homocysteine predicts progression of atherosclerosis. Atherosclerosis.

[23] Taylor LM, Moneta GL, Sexton GJ (1999). Prospective blinded study of the relationship between plasma homocysteine and progression of symptomatic peripheral arterial disease. J Vasc Surg.

[24] Graham IM, Daly LE, Refsum HM (1997). Plasma homocysteine as a risk factor for vascular disease. The European Concerted Action Project JAMA.

[25] Oudi ME, Aouni Z, Mazigh C (2010). Homocysteine and markers of inflammation in acute coronary syndrome. Exp Clin Cardiol.

[26] Fu Z, Qian G, Xue H (2015). Hyperhomocysteinemia is an independent predictor of long-term clinical outcomes in Chinese octogenarians with acute coronary syndrome. Clin Interv Aging.

[27] Yurtdas M, ÖzcanI T, Sabri AS (2013). Plasma homocysteine is associated with ischemic findings without organic stenosis in patients with slow coronary flow. J Cardiol.

[28] Tang O, Wu J, Qin F (2014). Relationship between methylenetetrahydrofolate reductase gene polymorphism and the coronary slow flow phemonenon. Coron Artery Dis.

[29] Sezgin N, Barutcu I, Sezgin AT (2005). Plasma nitric oxide level and its role in slow coronary flow phenomenon. Int Heart J.

[30] Sezgin AT, Topal E, Barutcu I (2005). Impaired left ventricle filling in slow coronary flow phenomenon: an echo-doppler study. Angiology.

[31] Tanriverdi H, Evrengul H, Enli Y (2007). Effect of homocysteine-induced oxidative stress on endothelial function in coronary slow-flow. Cardiology.

[32] Clarke R, Daly L, Robinson K (1991). Hyperhomocysteinemia: an independent risk factor for vascular disease. N Engl J Med.

[33] Glueck CJ, Show P, Lang JE, Tracy T, Sieve-Smith L, Wang Y (1995). Evidence that homocysteine is an independent risk factor for atherosclerosis in hyperlipidemic patients. Am J Cardiol.

[34] Celermajer DS, Sorensen K, Ryalls M (1993). Impaired endothelial function occurs in the systemic arteriesof children with homozygous homocystinuria but not in their heterozygousparents. J Am CollCardiol.

[35] Undas A, Brozek J, Jankowski M, Siudak Z, Szczeklik A, Jakubowski H (2006). Plasma homocysteine affects fibrin clot permeability and resistance to lysis in human subjects. Arterioscler Thromb Vasc Biol.

[36] Sauls DL, Wolberg AS, Hoffman M (2003). Elevated plasma homocysteine leads to alterations in fibrin clot structure and stability: implications for the mechanism of thrombosis in hyperhomocysteinemia. J Thromb Haemost.

[37] Lauricella AM, Quintana IL, Kordich LC (2002). Effects of homocysteine thiol group on fibrin networks: another possible mechanism of harm. Thromb Res.

[38] Quintana IL, Oberholzer MV, Kordich L, Lauricella AM (2011). Impaired fibrin gel permeability by high homocysteine levels. Thromb Res.

[39] Lauricella AM, Quintana I, Castañon M (2006). Influence of homocysteine on fibrin network lysis. Blood Coagul Fibrinolysis.

[40] Speidl WS, Nikfardjam M, Niessner A (2007). Mild hyperhomocysteinemia is associated with a decreased fibrinolytic activity in patients after ST-elevation myocardial infarction. Thromb Res.

